# P-1947. Aspergillus Susceptibility Trends in High-risk Hematologic Malignancy Patients with Pulmonary Infections

**DOI:** 10.1093/ofid/ofaf695.2115

**Published:** 2026-01-11

**Authors:** Lylybell Y Zhou, Rajshri Joshi, Ana Velez, Ju Hee Katzman

**Affiliations:** University of South Florida, Morsani College of Medicine, Tampa, FL; University of South Florida, Tampa, Florida; University of South Florida, Tampa, Florida; University of South Florida / Moffitt Cancer Center, Tampa, Florida

## Abstract

**Background:**

*Aspergillus* causes invasive fungal infections (IFI) in immunocompromised patients. Our center primarily uses voriconazole for prophylaxis for *Aspergillus*. Susceptibility patterns shift with prophylaxis/environmental factors. We assessed *in-vitro* susceptibility of invasive *Aspergillus* in high-risk hematologic cancer patients.

**Figure:**
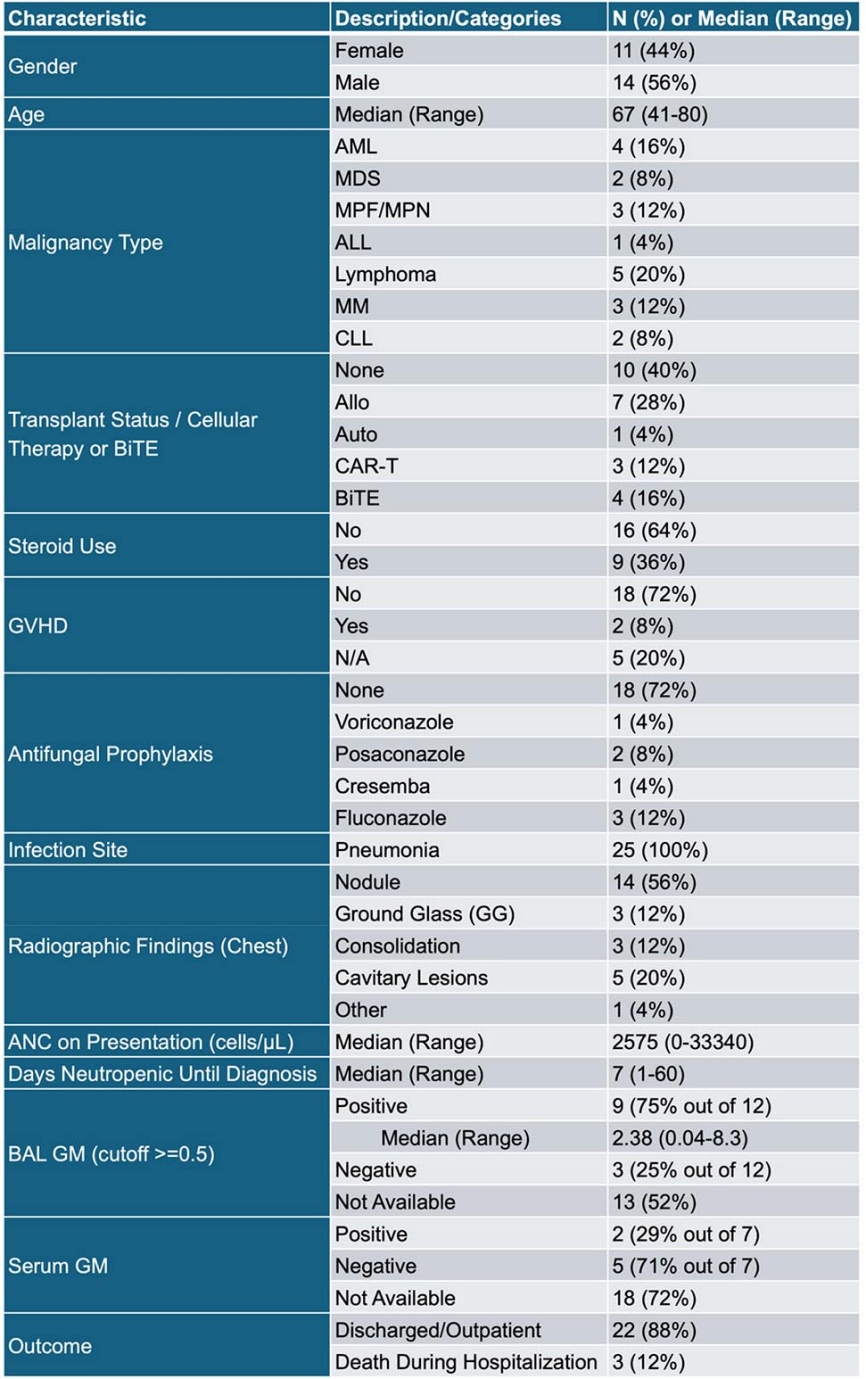
Clinical Characteristics and Outcomes of Patients with Aspergillus Infections (N = 25)

**Figure:**
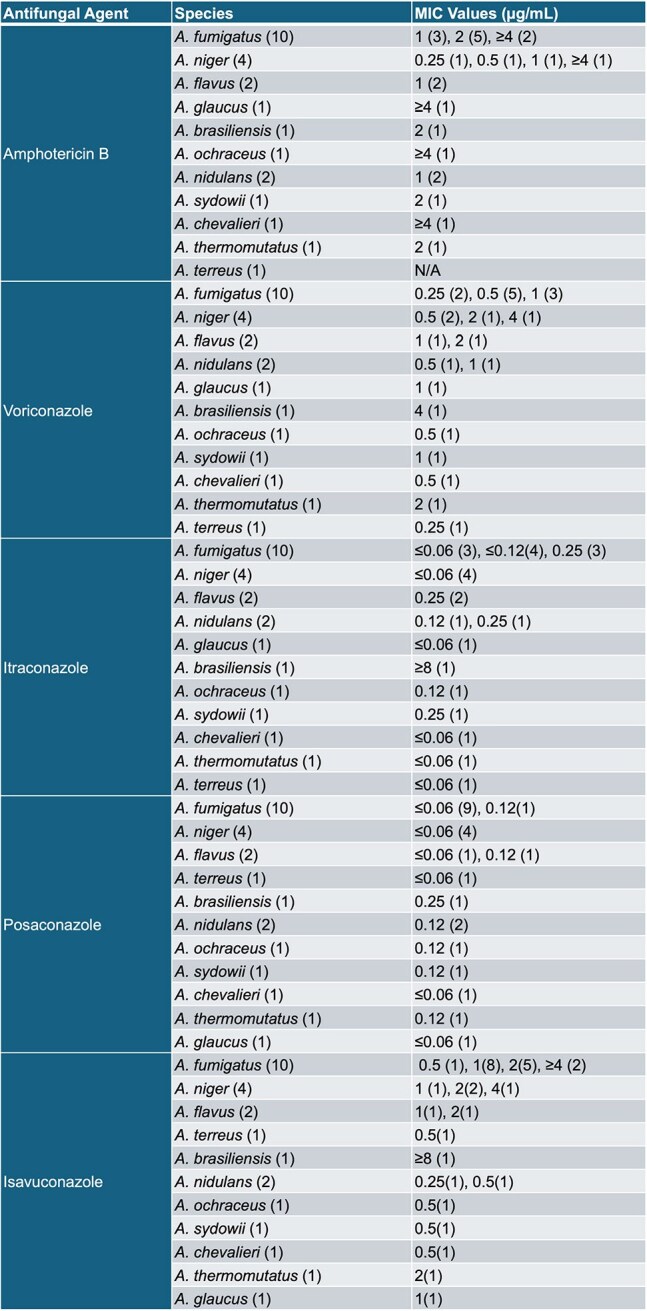
In Vitro Susceptibility of Aspergillus Species Isolates to Antifungal Agents (N=25)

**Methods:**

We performed a single-center MedMined chart review of hematologic malignancy patients with *Aspergillus* IFI (Jan 2023-Mar 2025), evaluating clinical/lab data and describe susceptibility.

**Figure:**
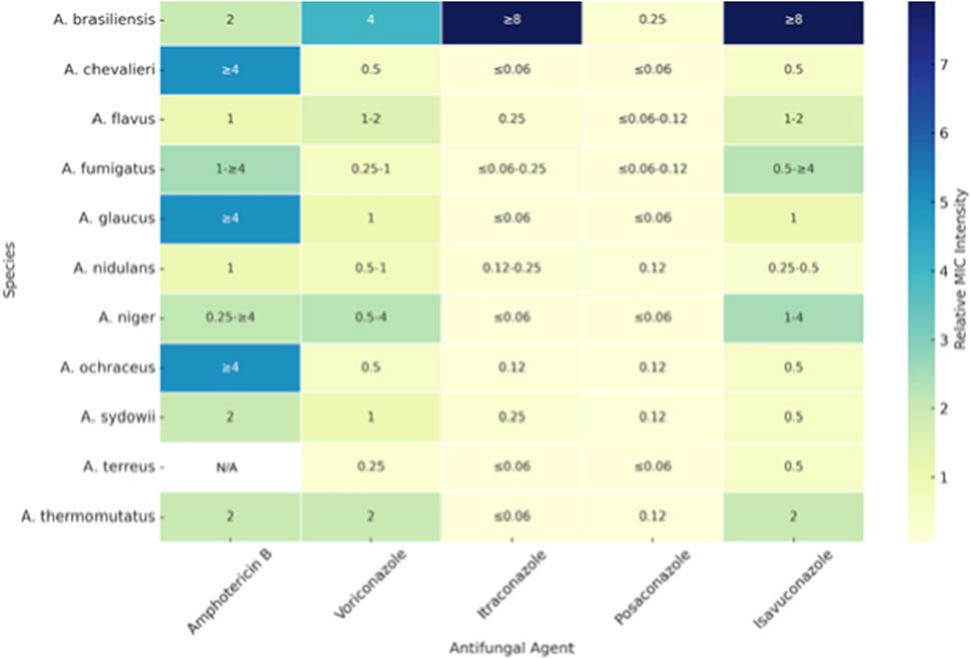
Minimum Inhibitory Concentration (MIC) Summary for Aspergillus Species Across five Antifungal Agents

This heatmap displays the susceptibility profiles of 11 Aspergillus species to five antifungal agents: Amphotericin B, Voriconazole, Itraconazole, Posaconazole, and Isavuconazole. MIC values are labeled within each cell using interpretative ranges (e.g., “≤0.06”, “1–≥4”) derived from in vitro susceptibility testing of clinical isolates. Background shading reflects the relative MIC intensity, where:

- Lighter shades indicate lower MIC values (greater fungal susceptibility)
- Darker shades indicate higher MIC values (reduced susceptibility or resistance)

MIC values are expressed in µg/mL. Cells marked as “N/A” represent species-drug combinations for which no data were available. This representation helps visualize and compare antifungal susceptibility trends across different Aspergillus species.

**Results:**

144 culture-positive cases were reviewed, 23 patients with probable IFI and hematologic malignancy were included; two had multiple *Aspergillus* species, which were included. We excluded 100 cases with solid tumors and 19 without susceptibility testing/deemed colonized. The specimen sources were BAL, sputum, blood, and tissue. Most patients (72%) were not receiving prophylaxis; 28% were neutropenic (ANC < 500) at diagnosis. All had pulmonary IFIs; 56% had nodular pneumonia. Of 12 patients with BAL galactomannan, 75% were positive (median 5.77 index, range: 0.04-8.3). In this cohort, MIC values were deemed susceptible for posaconazole [S: < =0.25] in all isolates, while isavuconazole [S: 1] was at 18 (72%), voriconazole [S: 0.5] was at 14 (56%), and amphotericin B [S: 1] was at 11 (44%). Mann-Whitney U tests showed no significant MIC distribution differences between discharged/deceased patients for amphotericin B (U=7.00, p=0.096), isavuconazole (U=12.000, p=0.244), posaconazole (U=20.000, p=0.721), or voriconazole (U=8.000, p=0.111).

**Conclusion:**

While all isolates showed low MICs and were deemed susceptible to posaconazole, a smaller proportion of isolates were considered susceptible to isavuconazole (72%), voriconazole (56%), and amphotericin B (44%). The death group tend to have higher amphotericin B MIC values (U=7.000, p=0.096). The increasing number of *Aspergillus* species with elevated MIC to voriconazole, isavuconazole, and amphotericin in this small high-risk patient sample is highly concerning. Further multi-center research is needed to validate these findings and identify antifungal resistance mechanisms in this population.

**Disclosures:**

All Authors: No reported disclosures

